# Building capacity through open approaches: Lessons from developing undergraduate electrophysiology practicals

**DOI:** 10.12688/f1000research.51049.1

**Published:** 2021-03-08

**Authors:** Erin C. McKiernan, Lucía Medina Gómez

**Affiliations:** 1 Departamento de Fisica, Facultad de Ciencias, Universidad Nacional Autónoma de México, Ciudad de México, CDMX, 04510, Mexico

**Keywords:** biomedical sciences, electrophysiology, higher education, undergraduate education, open education, open research, open scholarship, open science

## Abstract

**Background: **Electrophysiology has a wide range of biomedical research and clinical applications. As such, education in the theoretical basis and hands-on practice of electrophysiological techniques is essential for biomedical students, including at the undergraduate level. However, offering hands-on learning experiences is particularly difficult in environments with limited resources and infrastructure.

**Methods: **In 2017, we began a project to design and incorporate electrophysiology laboratory practicals into our Biomedical Physics undergraduate curriculum at the Universidad Nacional Autónoma de México. We describe some of the challenges we faced, how we maximized resources to overcome some of these challenges, and in particular, how we used open scholarship approaches to build both educational and research capacity.

**Results: **We succeeded in developing a number of experimental and data analysis practicals in electrophysiology, including electrocardiogram, electromyogram, and electrooculogram techniques. The use of open tools, open platforms, and open licenses was key to the success and broader impact of our project. We share examples of our practicals and explain how we use these activities to strengthen interdisciplinary learning, namely the application of concepts in physics to understanding functions of the human body.

**Conclusions: **Open scholarship provides multiple opportunities for universities to build capacity. Our goal is to provide ideas, materials, and strategies for educators working in similar resource-limited environments.

## Introduction

Electrophysiological techniques, like electromyogram (EMG), electrocardiogram (ECG), and electroencephalogram (EEG) recording, are commonly used in both clinical settings and biomedical research. For example, EMG recordings are used to study neuromuscular disorders ^1^ and spinal cord injury ^2, 3^; ECG recordings are used to detect cardiac conduction disorders ^4^ and heart attack ^5^; and EEG recordings are used to study epileptic seizures ^6, 7^ and sleep disorders ^8, 9^. Considering the importance of these techniques, it is vital that biomedical students receive training in their physiological basis, how to perform recordings, and how to analyze electrophysiological data, starting preferably at the undergraduate level.

As recently as a decade ago, several factors made doing electrophysiology with groups of students difficult if not prohibitive. Recording equipment was large, not portable, costly, and required expertise to operate. However, in recent years, companies have emerged dedicated to the production of low-cost but high-quality electrophysiology equipment, ideal for use in educational settings. For example, Backyard Brains (BYB; backyardbrains.com) is a company that designs and sells equipment to record action potentials (APs) in insects and plants, EMG and ECG in human subjects, and a variety of other electrophysiology products and accessories, most at prices below $300 U.S. dollars (USD). Many of these devices fit in the palm of your hand and connect to any smartphone, making them highly portable and easy to use. We have entered a new era when electrophysiology can now be easily brought into the classroom. However, many lesson plans and degree programs have yet to catch up.

In 2014, the Faculty of Science at the Universidad Nacional Autónoma de México (UNAM) – Latin America’s largest public university –launched its first undergraduate degree program in biomedical physics ^10, 11^. The overall goal of the program is to provide students with integrative theoretical and practical training in the areas of physics, mathematics, and biomedical sciences, to produce inter-disciplinary professionals that can work in diverse clinical and research environments. Specifically, the objectives of the program include, but are not limited to, educating students in: (1) physics applied to the study of the human body; (2) physics applied to medical diagnosis and therapy; and (3) physical principles underlying the instrumentation and function of the latest biomedical devices ^11^. We believe electrophysiology training is an important part of meeting these educational objectives. However, due to limited resources and infrastructure, none of our core courses previously included laboratory practicals in electrophysiology. The same limitations were also affecting our ability to develop electrophysiology research projects with our students.

In 2017 and 2019, we received funds through UNAM’s educational innovation grants (PAPIME) program to develop electrophysiology practicals for our biomedical physics students. With a total of nearly $17,000 USD over the last three years, we were able to buy recording equipment, microscopes, computers, instrumentation accessories, and more, and successfully developed electrophysiology practicals which we have released ( electrophys.wordpress.com and github.com/emckiernan/electrophys) as Open Educational Resources (OERs) ^12, 13^. Here we share examples of some of these practicals, their use in biomedical physics education, and how we integrated them into our curriculum. Furthermore, we describe the techniques and tools we used to make the most of the grant funds in a limited-resource environment, and specifically how open scholarship practices (open data, open education, open hardware, open protocols, open source) helped us broaden our impact and build not just educational but also research capacity. We hope sharing our experience will help other academics working in similar environments.

## Institutional context

To explain some of the motivation behind this project and its potential impact, it is important to first understand the environment in which we work, both within UNAM and the Faculty of Science.

### UNAM

UNAM is the largest public university in Latin America ^14^. As of 2019-2020, over 360,000 students were enrolled at UNAM, including more than 217,000 undergraduates and 30,000 graduate students ^15^. The university has 129 undergraduate and 41 graduate degree programs. Also, in 2019 UNAM served over one million students through its continuing education, including online, programs ^15^.

As a public insitution, education at UNAM is nearly free, subsidized by federal funds. Students pay an annual registration fee of just 20 Mexican cents (equivalent to ∼0.01 USD). This is combination with UNAM’s prestige and reputation for quality education results in a high demand for entry. Each year, less than 10% of applicants are accepted at the undergraduate level through UNAM’s admissions testing ^16^. In other words, a huge percentage of the eligible student population in Mexico is unable to study at this university that receives the largest share of public funds – an annual budget equivalent to approximately 2 billion USD ^17, 18^. One could argue that, more than any other public institution in Mexico, UNAM has a responsibility to give back to the community. On the other hand, while UNAM receives more funds than other public universities in Mexico, it still operates on a relatively limited budget considering its size and the number of services offered by the institution. For comparison, consider the University of California, which has a similar though smaller population of over 285,000 students ^19^ but almost 20 times the budget of UNAM ^20^. So, how can institutions like UNAM maximize the use of public funds, both for their benefit and that of the larger Mexican population?

### Faculty of Science

UNAM comprises 15 faculties, 34 institutes, and various other centers, schools, and units ^15^. There are fundamental differences for academics working in faculties versus institutes, which are important for understanding our work as professors in the Faculty of Science.

In institutes, the primary focus is research. Laboratory space is assigned to many faculty at the time of hiring and their teaching load is low. According to the UNAM Statute of Academic Personnel, researchers in institutes must teach a minimum of 3 contact hours per week each semester ^21^, equivalent to a 1-1 teaching load at Canadian or U.S. institutions. In contrast, faculties are focused on teaching. Entry-level professors are required to teach a minimum of 9 contact hours per week each semester ^21^, equivalent to a 3-3 teaching load. Unlike at many institutions in North America, there are no standard mechanisms for ‘buying out’ of teaching if a professor receives a grant. In addition, professors are expected to contribute significantly to ‘formation of human resources’ by directing student social service projects and theses, serving on committees, and tutoring. Many professors work almost exclusively with undergraduates, especially for the first few years when their professoriate level does not allow advising graduate students in many degree programs. Despite the heavy teaching and service load, there is still a research expectation. However, professors are not necessarily assigned laboratory space and receive no start-up funds. Availability of laboratory space in the Faculty of Science has become especially problematic as student and academic population growth puts increasing demands on an already overloaded infrastructure.

These conditions raise a number of questions for professors working in faculties like UNAM’s Faculty of Science: With limited resources and infrastructure, how do I provide high-quality, hands-on educational experiences for my classes?; how do I develop research projects for social service and thesis students?; and how do I build up my own research program and start producing?

## PAPIME educational grants

A partial answer to some of the above questions comes in the form of internal grants offered by the General Directorate for Academic Personnel Affairs (Dirección General Asuntos del Personal Académico; DGAPA) at UNAM. One of these grant programs – the Support Program for Projects to Innovate and Improve Education (Programa de Apoyo a Proyectos para Innovar y Mejorar la Educación; PAPIME) – focuses on education ^22^. This program has been key for us in building capacity and is a funding mechanism we believe more universities should emulate. The goal of PAPIME is to, “Promote the improvement and development of academic staff by supporting projects that lead to innovation and improvement of the teaching-learning process and benefit students...Teaching innovation projects should revolve around themes that allow creative teaching, with new ways of thinking, to motivate the interest and imagination of students” ^22^.

The 2020 call for applications ^23^ shows that these grants fund a wide range of projects and diverse products, including but not limited to: (1) teaching materials, like exercises or practicals, case design, tutorials, digital applications, software, and websites; (2) publications, like books or articles in areas such as educational research; (3) innovative educational evaluation systems, strategies, and instruments; (4) organization and participation in academic events, like colloquia and seminars; and (5) training activities, like in-person or online courses and workshops, or fieldwork.

These grants are typically 1 year in duration, and as of 2020 can be awarded up to $250,000 MXN annually ^23^, or ∼12,500 USD. Interestingly, while not explicitly using the language, PAPIME grants can function to a certain extent as OER grants. Products resulting from PAPIME projects are required to be uploaded to UNAM’s Repository of Educational Innovation (Repositorio de Innovación Educativa, RIE: innovacioneducativa.unam.mx). Digital materials in particular must be uploaded to UNAM’s University Learning Network (Red Universitaria de Aprendizaje, RUA: rua.unam.mx). The stated objective of sharing these materials is to “disseminate and extend coverage for the benefit of the university community and thus optimize the resources invested by UNAM in development of the project” ^23^. In line with this, projects are evaluated on several characteristics related to broadness of impact, including: (1) number of students that will benefit from the project; (2) where students come from, whether inside the academic entity, university, or beyond; (3) number and names of classes that will benefit from the materials; and (4) number of professors that will use the products. Using an open approach can help academics argue broader impact, i.e. a larger population of both students and educators are reached, within and beyond the institution, and materials can be reused, revised, remixed, and redistributed ^12^.

## Electrophysiology grant

We were awarded our first PAPIME grant in 2017 and our second in 2019. The idea for the overall project came from what we perceived to be a gap in the education of our Biomedical Physics undergraduates, namely a lack of hands-on training in electrophysiology and related skills. We set out to design electrophysiology laboratory practicals that could be incorporated into our plan of study. Not all these practicals were intended to be 100% novel; resources exist on the basics of EMG ^24, 25^ and ECG ^26, 27^ recording, for example. BYB has already developed over 60 experiments that can be performed using their equipment and released these on their website ( backyardbrains.com/experiments) as OERs under an open license. However, there are a few ways we wanted to expand and extend existing work.

First, we wanted all our practicals to be accompanied by more in-depth lesson plans. The BYB tutorials are excellent starting points, but are too simple for our fourth-semester human physiology undergraduates (e.g., their EMG tutorial ^24^ is marked as ‘beginner’ for elementary school students 5th grade and up). On the other hand, many of the resources we found in the scientific literature were too complicated, aimed more at graduate students or working professionals (e.g., 25). In addition, many of these latter resources have a clinical rather than biophysics focus. We saw a need for electrophysiology OERs designed for a more intermediate, undergraduate level that would reinforce material seen in our core courses, including physics as applied to the human body.

Second, we aimed to develop novel practicals that would combine electrophysiology with other physiological measurements like spirometry, helping our students see how different systems in the body work together. Currently, as in many universities, our human physiology course is taught as a sequence of system-based modules (e.g., nervous system, cardiovascular system, etc.). As Conford ^28^ writes, “One assumption that many modular courses presently reflect is that effective learning proceeds via self-contained chunks of information...Modules, however, by their very structure, tend to fragment knowledge rather than to integrate it” (pg. 243). We see these practicals as a way to recover this integration and connect concepts across modules.

Third, we sought to develop bilingual materials. We have struggled to find quality Spanish-language OERs, especially in biophysics. Language can be a significant barrier to OER reuse and remixing ^29– 32^. From an UNESCO report ^33^, “Not only does the English language dominate OER provision, but English-language content tends to be based on Western learning theory. This limits the relevance and accessibility of OER materials in non-English, non-Western settings. There is a risk that language barriers and cultural differences could consign less developed countries to the role of OER consumers rather than contributors to the expansion of knowledge” (pg. 12).

Finally, we wanted to develop a suite of products around each practical and release not just written OERs but also accompanying code, data, images, and videos, all under open licenses. We reasoned this was one important way to increase the impact of the project. For example, educators without the resources to buy recording equipment could at least reuse our data and code to graph and analyze electrophysiology recordings with their students.

## Building capacity

With our first grant in 2017, we were awarded the equivalent of ∼$10,500 USD. We used the bulk of the funds to purchase electrophysiology recording equipment ( Table 1), microscopes, and related accessories like electrodes and dissection tools ( Table 2). The remaining funds were used to finance scholarships for two undergraduates to work on the project. With our second grant in 2019, we were awarded ∼$6,300 USD that we used to purchase computer equipment, surface electrodes for recording, instrumentation accessories like Raspberry Pi 3 Model B and Module V2 cameras, Arduino sensor kits, and food and bedding for experimental animals. (Many of these are standard products available from multiple providers, so we did not itemize these in table form). We also gave scholarships to two more undergraduates. While the amounts awarded us may sound sizeable – similar OER grants in the U.S. and Canada often cap at $5,000 USD ^34– 36^ – this was still a limited budget considering we were starting from zero in terms of equipment and materials. We had to maximize use of these funds to build capacity.

**Table 1.** Electrophysiology equipment purchased.

item	purpose	vendor	price *	units	total	total
Neuron SpikerBox	Bundle record APs from insects like cockroaches or crickets	Backyard Brains	$99.99	3	$299.97	backyardbrains.com/products/spikerboxBundle
DIY Neuron SpikerBox	kit to build Neuron SpikerBox	Backyard Brains	$49.99	3	$149.97	backyardbrains.com/products/diyspikerbox
Neuron SpikerBox Pro	two channels for dual recordings of APs	Backyard Brains	$229.99	2	$459.98	backyardbrains.com/products/neuronspikerboxpro
DIY Neuron 2-Channel SpikerBox	kit to build 2-channel Neuron SpikerBox	Backyard Brains	$99.99	3	$299.97	backyardbrains.com/products/diytwochannel
Muscle SpikerBox Bundle	record EMGs from skeletal muscles in human subjects	Backyard Brains	$149.99	3	$449.97	backyardbrains.com/products/muscleSpikerboxBundle
DIY Muscle SpikerBox	kit to build Muscle SpikerBox	Backyard Brains	$79.99	3	$239.97	backyardbrains.com/products/diyMuscleSpikerbox
Muscle SpikerBox Pro	record dual channel EMGs from pairs of skeletal muscles in human subjects	Backyard Brains	$249.99	2	$499.98	backyardbrains.com/products/musclespikerboxpro
Muscle SpikerShield Bundle	interface to control simple prosthetics with muscle contractions	Backyard Brains	$149.99	2	$299.98	backyardbrains.com/products/muscleSpikershieldBundle
DIY Muscle SpikerShield	kit to build Muscle SpikerShield	Backyard Brains	$64.99	2	$129.98	backyardbrains.com/products/diyMuscleSpikerShield
Heart and Brain SpikerBox	record ECG, electrooculogram (EOG), or simple EEG in human subjects	Backyard Brains	$149.99	2	$299.98	backyardbrains.com/products/heartAndBrainSpikerBox
Plant SpikerBox	record APs in plants	Backyard Brains	$149.99	1	$149.99	backyardbrains.com/products/plantspikerbox

*All prices in USD. Prices at time of purchase, not including shipping and handling, taxes, etc.

### Electrophysiology equipment

All electrophysiological recording equipment was obtained from BYB ( backyardbrains.com), including devices to record APs in insects (Neuron SpikerBox), and EMGs (Muscle SpikerBox) or ECGs (Heart and Brain SpikerBox) in human subjects ( Table 1). We purchased these as bundles, which included the recording device, cables, surface electrodes, conductive gel, and other accessories. The low cost of these bundles ( *<*$250 USD each), compared to conventional electrophysiology equipment, allowed us to purchase multiple devices. With 3 or 4 devices, we could work in groups of 5-10 and pilot practicals with classes of 20–30 students.

We also purchased DIY kits from BYB to build additional recording devices. This served two purposes. First, students will assemble the devices, learning valuable instrumentation skills in the process, which is one of the core objectives of our Biomedical Physics plan of study. Students will fully document the assembly process with step-by-step protocols, photos, and videos, which will be shared online as OERs. Second, at around half the price of the fully assembled device bundles, DIY kits allowed us to buy more equipment without exceeding our budget. Once assembled, our recording capacity will double, meaning we can work with more students.

Affordability was not the only advantage of the BYB equipment. The small size and portability of the devices meant we did not need a dedicated laboratory space, solving one of our infrastructure issues. We could take these devices into any classroom and record with students using their smartphones. We also allow students to borrow these devices and take them home to work on individual research projects. A few years ago, having students do electrophysiology at home would have been impossible. Now we can offer them this unique experience, which can be a huge motivating factor in their academic development. Since 2017, students and professors in our program have used the equipment in core and elective coursework, social service projects, and thesis research. In other words, purchasing a small amount of equipment has greatly increased our capacity to provide high-quality educational and research opportunities for our undergraduates.

### Electrophysiology accessories

We purchased several accessories to improve both recording experiences for students and potentially research capacity ( Table 2). For example, while the BYB electrodes that come with the Neuron SpikerBox are sufficient for basic AP recording in large insects, they are stainless steel sewing needles with a relatively large tip diameter (0.25–0.6 mm) ^37^, non-insulated, and not ideal for finer recordings in smaller preparations or cells. So, we purchased insulated Tungsten electrodes with a 2–3 *µ*m tip diameter. At ∼$19 USD each, these electrodes are only $9 more than BYB’s, but should provide a substantial improvement in recording capabilities and quality. For less than $200 USD we can buy a packet of 10 Tungstens and upgrade 10 SpikerBoxes.

**Table 2.** Other equipment and accessories purchased.

item	purpose	vendor	price *	units	total	link
Arduino kit	instrumentation with Arduino Uno; includes variety of motors, sensors, resistors, LEDs, etc.	SIET México	$71.69	10	$716.90	no product webpage
Sensor kit	sensors (temperature, touch, sound) and accessories (buzzers, joysticks, switches) for instrumentation	SIET México	$68.31	3	$204.93	no product webpage
Advanced Zoology Dissecting Set	fine dissection, extracting brain tissue; 24-piece set	VWR via DICONSS	$45.26	2	$90.52	tinyurl.com/yye4kz7g
Classroom dissection set	dissection and manipulation of small preparations for recordings; 261- piece set for 20 students	VWR via DICONSS	$204.74	1	$204.74	tinyurl.com/yyb9f2hp
High Power RoachScope	small, portable microscope for use with smartphone to visualize preparations for recording	Backyard Brains	$99.99	3	$299.97	backyardbrains.com/products/roachscope
Illuminated pocket microscope	small, portable microscope for use in classroom or fieldwork	Fisher Scientific	$48.58	1	$48.58	tinyurl.com/y4g3mmyc
Stereomicroscope	visualization and dissection of small preparations, magnification for instrumentation	Fisher Scientific	$291.63	3	$874.89	tinyurl.com/y45tqpsr
Trinocular microscope with digital camera	visualization of samples at high magnification; camera to connect to display monitor and photograph samples	National Optical via Fisher	$1825.11	1	$1,825.11	tinyurl.com/y4pyzjdw
Manipulator	position and move electrodes with control and precision	Backyard Brains	$99.99	3	$299.97	backyardbrains.com/products/micromanipulator
Tungsten electrode	fine-tip electrodes for recordings or stimulation; package of 10	WPI via Alta Tecnología en Laboratorios	$189.47	1	$189.47	tinyurl.com/y4plmcuu

*All prices in USD. For items purchased in MXN, an exchange rate of 19 pesos to the dollar was used to estimate amounts. Prices at time of purchase, not including shipping and handling, taxes, etc.

We also purchased manipulators to improve control and precision of electrode placement. Conventional 3-axis manual micromanipulators, like those made by Narishige ( usa.narishige-group.com), cost ∼1,000 USD and were out of our price range. However, BYB provides a 3-D printed plastic manipulator with 3 axes of movement in the millimeter range and adjustable electrode angle through 135 degrees for just $99.99. Furthermore, BYB’s open hardware approach means the plans for printing and building the manipulators are available on their website, which will allow us to reduce costs in the future by printing more manipulators at a university facility. In fact, the growing open labware/maker movement is increasingly allowing researchers to 3-D print their own lab equipment, including electrophysiology devices and accessories, for a fraction of the cost ^38, 39^.

We also bought accessories from a local provider (SIET México), including Arduino kits, sensor kits, Raspberry Pi 3 Model B, and Raspberry Pi Cameras Module V2. Arduino kits include an Arduino Uno R3, servo and step motors with drivers, a variety of sensors (infrared, humidity, temperature), and other accessories such as cables, resistors, and LEDs. Sensor kits are designed to be used in conjunction with Arduinos, and include heartbeat, temperature, touch, and sound sensors, as well as buzzers, joysticks, and switches. Similar Arduino and sensor kits can be purchased through Amazon or eBay. Kit components can be used for a variety of electrophysiology-related projects, including instrumentation of simple myoelectric prosthetic prototypes ^40, 41^.

### Dissection tools and microscopes

Electrophysiology often involves dissection to prepare tissues or cells for recording. Dissection tools, especially those for fine dissection, are costly. Fortunately, companies like VWR provide economic solutions in the form of classroom dissection sets, which include scissors, forceps, scalpels, pins, and more. With tools for up to 20 students and priced at _∼_$200 USD or less, the cost comes out to only _∼_$10 USD per student. With the remaining funds we had for tools, we bought just two fine dissection kits for use in more advanced, individual student projects.

Dissection and fine detail instrumentation also requires visualization and magnification. With this in mind, we purchased several microscopes with different characteristics. The Fisherbrand Illuminated Pocket Microscope weighs just 85 grams, measures 140L × 38W × 22H mm, and has 60–100x magnification. Similarly, the BYB High Power RoachScope weighs 400 grams, measures 142L × 94W × 74H mm, and can be used in combination with any smartphone camera. With digital zoom, it has 5–100x magnification. Both these microscopes are designed for maximum portability, so they can be taken into the classroom. In addition, both cost less than $100 USD each, so we could buy several to work with groups of students. We also purchased three Fisher Science Education Advanced Stereomicroscopes for just under $300 USD each. These microscopes are not very portable, but should give us better optics. To increase the utility of these microscopes, we are planning on 3-D printing a low-cost adapter that will attach to the eyepiece and allow us to mount any smartphone to take high-quality pictures or video. Open plans for such an adapter are available via the NIH 3D Print Exchange ^42^ and pictured in 39.

Finally, we purchased an advanced trinocular microscope (National Optical via Fisher Scientific) with 4x, 10x, 40x, and 100x objectives and a built-in digital camera (Moticam 1080 HDMI & USB) for high-resolution viewing of tissues and cells. The higher cost of this microscope meant we could only buy one. However, connecting the camera to a large computer monitor allows us to carry out demonstrations and have groups of students view samples simultaneously. We have also hosted “open house” events for new students using this microscope.

## Data acquisition and analysis

Commercial software used to record, process, and analyze electrophysiology data is often a significant expense for many laboratories. We did not have the budget to pay for software licenses, but also felt to do so would be incompatible with the open spirit of the project. It was important to us that any software we used be open source, and that any analysis code we created also be open to facilitate reuse. BYB provides the SpikeRecorder application free through their website ( backyardbrains.com/products -> Software) and source code via GitHub ( github.com/BackyardBrains/Spike-Recorder-IOS). The app can be downloaded and installed on students’ phones in minutes to begin recording. All recordings are saved as .wav audio files, which can then be played back, visualized, and some analysis performed within the same app ^43^. However, for analysis we felt we needed more control and customization, so we wrote code in Python (version 3.7.4) using the following packages: math, Matplotlib ^44^, NumPy ^45, 46^, os, pandas ^47^, random, SciPy ^48^, statistics, sys, wave, and wfdb. All our code is available via our GitHub repository at github.com/emckiernan/electrophys and archived on Zenodo at doi.org/10.5281/zenodo.4554420 ^49^.

Python code was developed inside Jupyter notebooks, which provide an interactive way to document and share code ^50, 51^. Our notebooks walk students through the process of opening and graphing recordings, applying filters, and quantifying aspects of electrical activity. The notebooks include exercises for students to perform in or outside of class as data analysis practicals. In other words, we create OERs out of this shared code ^52– 54^. As Downes ^53^ writes, use of Jupyter notebooks in this way “changes the conception of an educational resource from something static to something that’s interactive, to something that can be used to create, as well as to consume” (pg. 9). This also helps us meet another core learning objective of the Biomedical Physics plan of study, namely programming skills. In fourth semester, when students start with our electrophysiology practicals in their human physiology course, they also take a programming course which primarily teaches Python. Data analysis practicals are a good way for them to apply new programming skills to biomedically relevant data analysis, and integrate knowledge from these two core courses.

## Workflow and related tools

The development of most of our practicals began as a free-form process. We had a general idea of the type of recording we wanted to perform, and piloted these ideas first with classes of 20–30 undergraduates. Students were encouraged to experiment, for example by trying out different electrode placements and exercises. Subsequently, students wrote individual reports with background information, protocols, results, and conclusions. Students shared their photographs, videos, data, and reports with us via Google Drive. From the resources provided by students, we collected the best examples and used this information to build our master documents for each practical. In addition, four students were given scholarships with PAPIME funding to help us run pilots, gather materials, analyze data, and draft protocols. In this way, students played an active role in OER development, reinforcing Buckland’s idea of “students as content creators” ^55^.

Our master documents were written in LaTeX using the Overleaf platform ( overleaf.com). LaTeX presents a variety of advantages over word processing software, including control over document layout and figure placement, excellent equation handling, and automatic reference formatting ^56^. The Overleaf platform in particular provides several benefits. First, basic accounts are free, so there were no additional costs, as would be incurred by using commercial packages like Microsoft Office. Second, we could easily share and collaborate on a master file with integrated commenting functions and version control. Finally, Overleaf provides a rich text viewing option, which is more user-friendly, especially for undergraduates just starting out with LaTeX.

Once we had final versions of the master documents, these were uploaded to a public repository on GitHub ( github.com/emckiernan/electrophys), along with images, data, and code associated with each practical. GitHub provides Git version control ^57, 58^, which means OERs can continue to evolve as necessary while preserving the history of resource development ^59^. GitHub also provides collaboration features, which we hope students and educators will use to improve and customize these materials. However, we recognize not everyone uses GitHub, and that only hosting our materials there could represent a barrier to reuse. So, we built a Wordpress website to share materials in a more user-friendly way. This was done by converting our LaTeX documents to html using Pandoc ( pandoc.org), and then copying the html to a free Wordpress template ( wordpress.org). Minor formatting to improve visual presentation was done by hand. Jupyter notebooks were uploaded by creating public gists ( gist.github.com) and then copying these links to the Wordpress site for embedding. Using the free Wordpress services meant we did not incur any costs for website creation or hosting.

**Figure 1.**
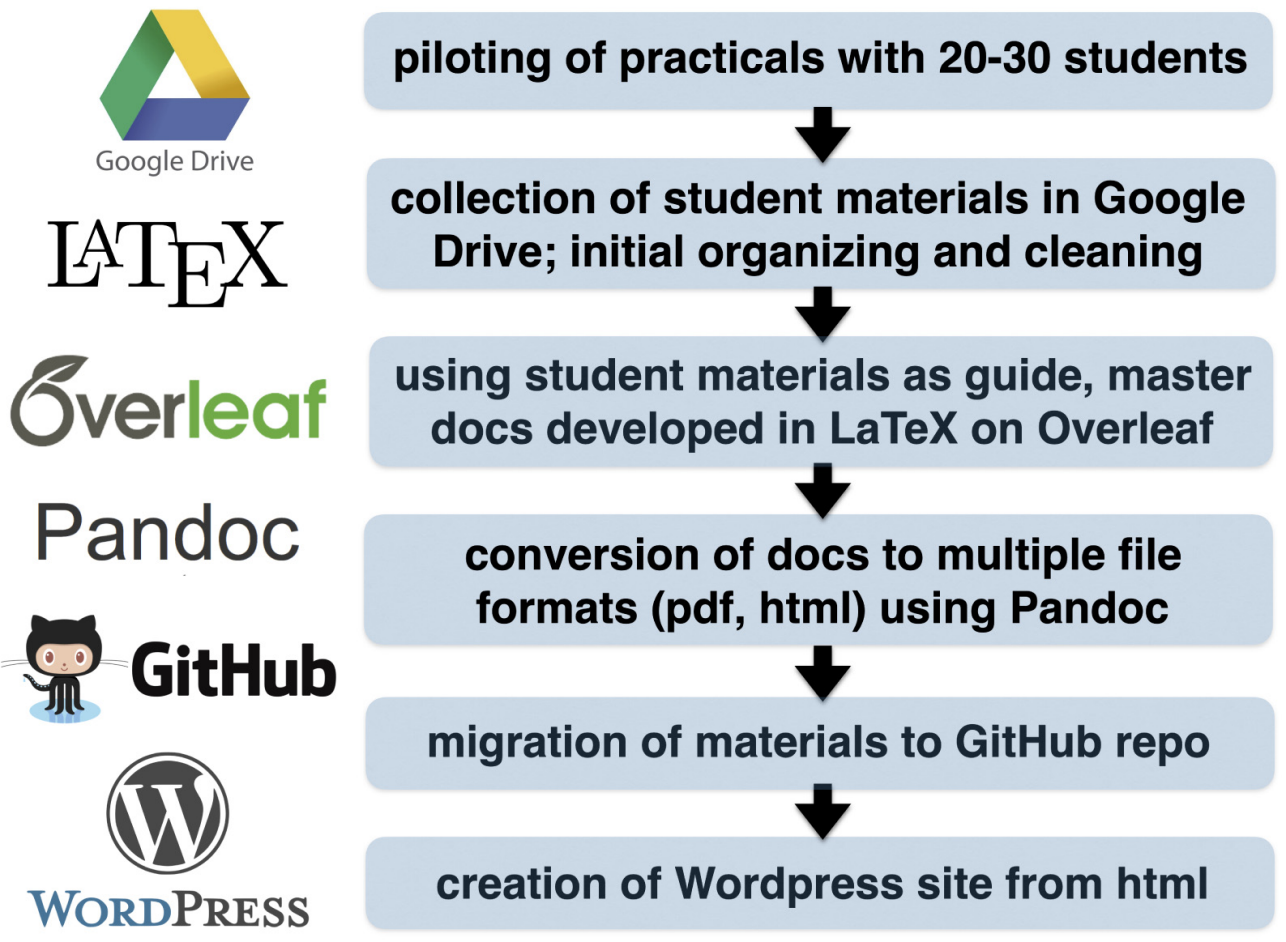
Workflow and tools used to pilot, develop, and share our electrophysiology practicals.

Our workflow is visualized in Figure 1. Moving forward, there are ways we could improve this workflow. For example, a more efficient way to set up our website would be to use GitHub Pages ( pages.github.com). This would allow for automatic syncing of the website when the repository materials are updated, but requires more in-depth html knowledge to properly format and maintain the site. We would also like to explore open source alternatives to several of the tools we used. Bosman and Kramer outline a potential open science workflow (as well as other workflows ranging from traditional to experimental) that could be useful for researchers and educators ^60^. More information on tools, including some open source alternatives, is available in Table 3.

**Table 3.** Open scholarship tools and platforms useful for teaching and student advising.

	tool/platform	link	advantages	possible uses
**Document preparation** **and collaborative** **writing**				
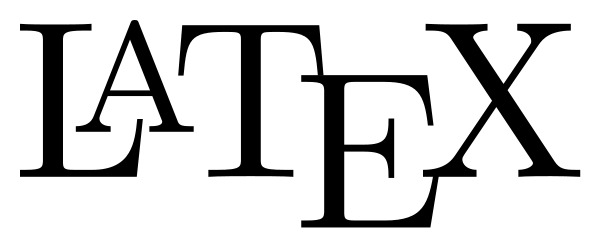	LaTeX	latex-project.org	customizable typsetting, great equation handling, automatic reference formatting and updating ^56^	document preparation for student reports, theses, and articles
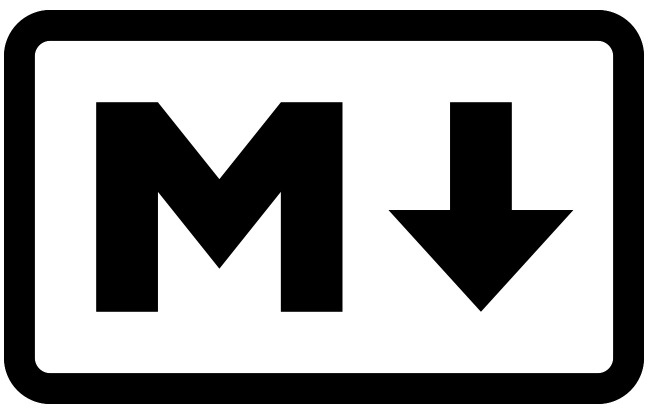	Markdown	markdownguide.org	’lightweight’, simple typsetting; easy conversion to multiple file formats; this flexibility allows transforming documents into OERs ^61^	student reports, adding text to Jupyter notebooks; e-books or website creation
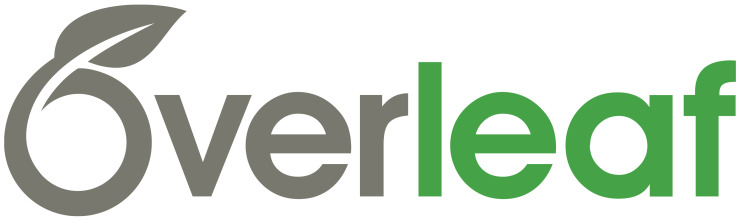	Overleaf	overleaf.com	collaborative online writing in LaTeX; richtext mode; commenting feature; Git version control; templates; direct submission to preprint servers and journals	hosting and collaborating on student reports, theses, articles; advisers can track progress and leave comments; student has backup with versioning
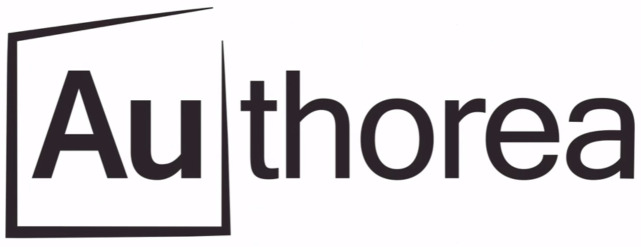	Authorea	authorea.com	collaborative online writing in LaTeX or Markdown; Git version control, in-platform publishing with DOI or submission to journals; multimedia file hosting	similar uses to those for Overleaf; ideal if wanting to embed data in report
**Repositories with** **version control for** **code/data sharing**				
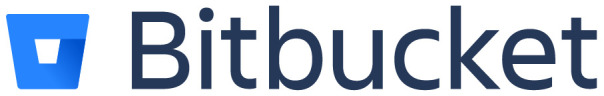	Bitbucket	bitbucket.org	version control with Git or Mercurial; collaborative features; wikis for project documentation; free unlimited private repositories for up to 5 collaborators	code and data sharing for student projects, especially if we want private repositories while materials are in development
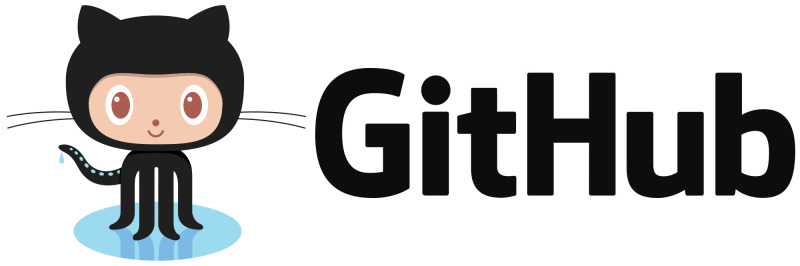	GitHub	github.com	version control using Git; collaborative features; wikis; free unlimited private repositories for up to 3 collaborators; large online community	similar uses to those for BitBucket; large online community could mean more eyes on our project and more collaborators
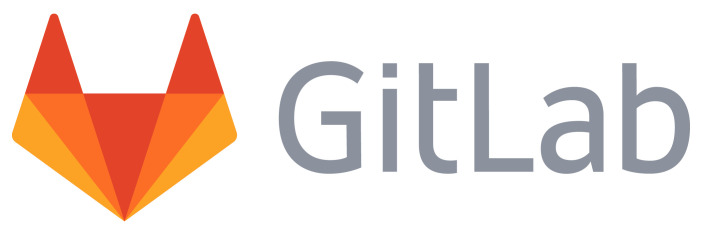	GitLab	about.gitlab.com	version control using Git; collaborative features; wikis; free private repositories with unlimited collaborators; open source	similar uses to those for BitBucket and GitHub; ideal if wanting a fully open source solution
**Repositories for sharing** **figures, posters,** **protocols**				
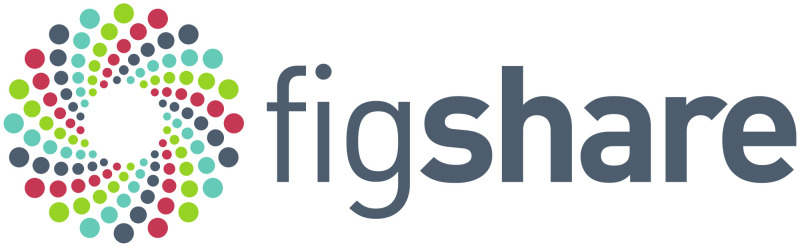	Figshare	figshare.com	accepts diverse scholarly products; many file formats; citable DOI; integration with other services, like GitHub and Overleaf	sharing student work like figures from theses or posters from symposia
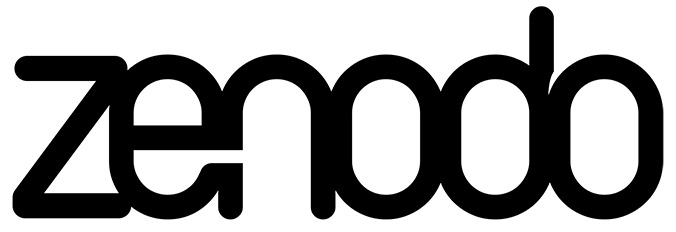	Zenodo	zenodo.org	accepts diverse scholarly products; many file formats; citable DOI; flexible licensing; integration with other services, including GitHub; open source	similar uses to those for Figshare; ideal if wanting an open source solution
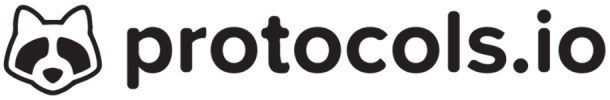	Protocols.io	protocols.io	share private or public step-by-step protocols; citable DOI; commenting and collaborative features; versioning	sharing and collaborating on protocols for lab classes or thesis projects; e.g. a protocol ECM published with students ^62^
**Data analysis and** **mathematical modeling**				
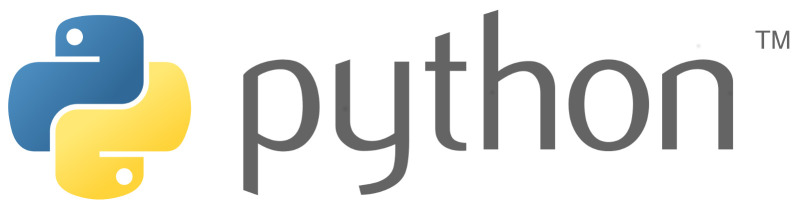	Python	python.org	versatile programming language; libraries for scientific computing and analysis; large online community; open source	programming courses; coding exercises for class demonstrations or practicals; analysis or modeling for student projects and theses
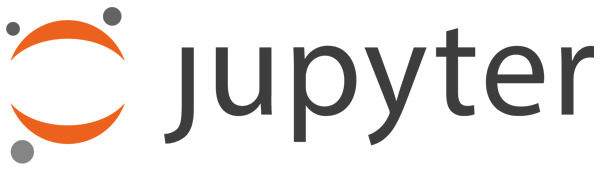	Jupyter	jupyter.org	interactive way to share code in multiple languages, including Python; include text to explain code; can convert notebooks to multiple file formats	interactive teaching; creating class exercises and data analysis practicals; documenting and sharing code for student research projects and theses
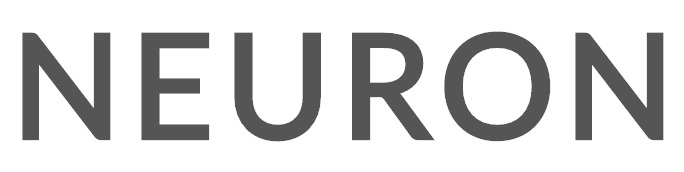	Neuron	neuron.yale.edu	simulation environment for modeling neurons with morphology or networks; graphical user interface; library of biophysical features and analysis tools; open source	class demonstrations and practicals; student projects and theses; study how electrical current spreads in cells; incorporate data
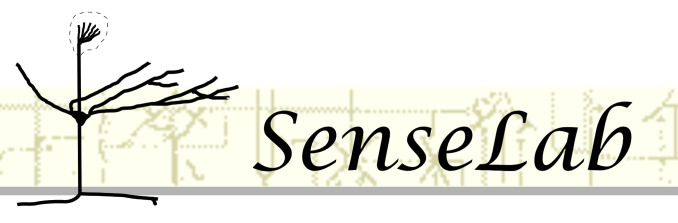	SenseLab	senselab.med.yale.edu	portal to databases with models of neurons and networks; models in multiple languages, including Python and Neuron	learning about model contruction and incorporating electrophysiology data; student projects, e.g. pick model, recreate then modify it
**Electrophysiology**				
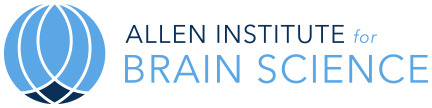	Cell types database	celltypes.brain-map.org	electrophysiological data from human and mouse neurons; openly licensed; open source analysis and modeling tools	exploring and analyzing data for class exercises, practicals, or student projects; teach students to recognize and interpret different types of electrical activity
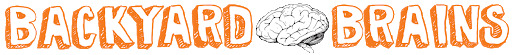	Backyard Brains	backyardbrains.com	product pages include open schematics; some include open code; bank of over 60 experiments with written tutorials and videos; teacher guides	teaching circuit construction, instrumentation; carrying out experiments introduces students to electrophysiology and generates ideas for new projects
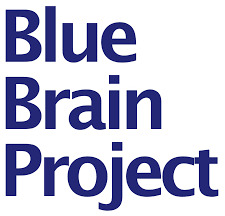	Channelpedia	channelpedia.epfl.ch	ion channel data, including gene expression, functions, and mathematical models; electrophysiology recordings, free download; community contributions	teaching students how different ion channels affect electrical activity; reuse data for analysis practicals and student research projects
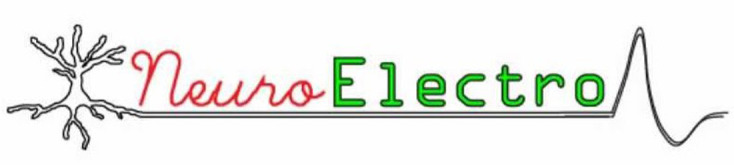	NeuroElectro	neuroelectro.org	database of electrophysiological properties of neurons compiled from literature; search by neuron type or property; free data download	teaching about variability in electrophysiological properties; students can use data to get parameter ranges for models
**Instrumentation**				
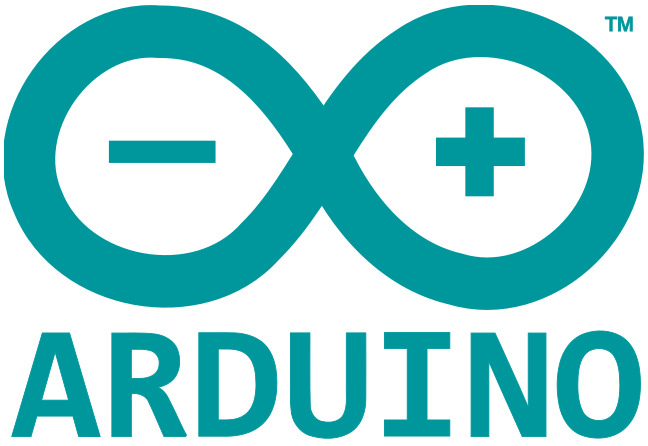	Arduino	arduino.cc	low-cost, versatile programmable microcontroller; interfaces with many sensors and devices; open hardware and open source software; online community	variety of simple to complex electrophysiology instrumentation projects, like building EMG recording devices or simple myoelectric prosthetics ^40, 41^
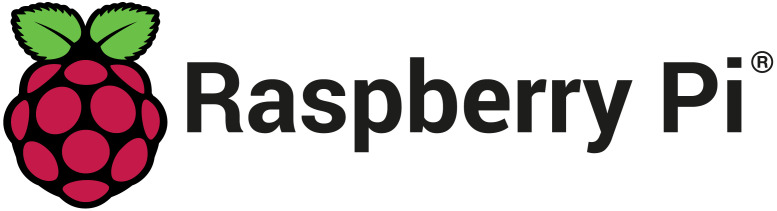	Raspberry PI	raspberrypi.org	low-cost, versatile single-board computer; small, portable; USB, HDMI, SD ports; free software; online community	variety of simple to complex electrophysiology instrumentation projects; can interface with Arduino
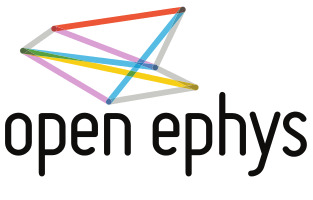	Open Ephys	open-ephys.org	information on how to build and use open hardware tools; community contributions; sell low-cost hardware for electrophysiology instrumentation ^63^	teaching students how to build electrophysiology recording devices; buying hardware to build more advanced devices for student research projects
**Website creation and** **hosting**				
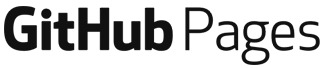	GitHub Pages	pages.github.com	automatic syncing between GitHub repository and website; available themes; customizable URLs; free hosting	building websites for classes, student research projects, or lab groups; online hosting of ebooks and other OERs
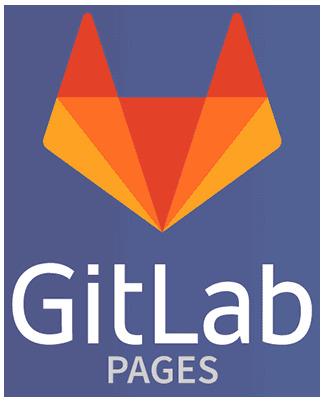	GitLab Pages	about.gitlab.com/ stages-devopslifecycle/ pages/	automatic syncing between GitLab repository and website; works with multiple site generators and plugins; tool integration; free hosting	uses similar to GitHub Pages; ideal if wanting an open source solution
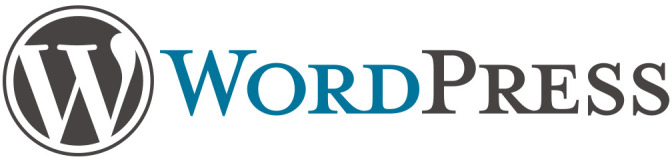	Wordpress	wordpress.com	user-friendly interface for site building; large bank of free templates; variety of plugins; site statistics; free hosting	uses similar to GitHub/GitLab Pages; ideal if little experience with git and html
**Publishing student** **work or educational** **resources**				
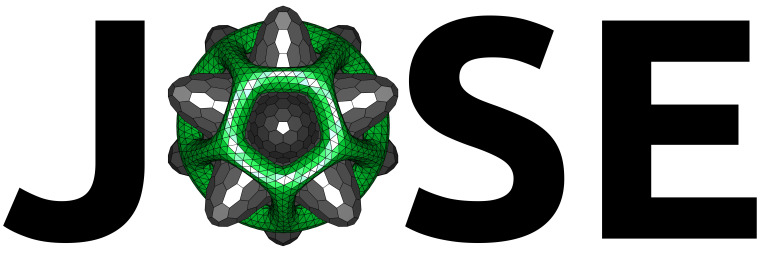	Journal of Open Source Education	jose.theoj.org	open access journal; publishes descriptions of open source educational materials, including software; focus more on products than paper; no fees for authors	getting credit, in the form of publication, for development of educational software, like a suite of Jupyter tutorials; diffusion and visibility for software OERs
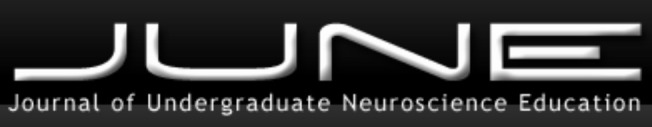	Journal of Undergraduate Neuroscience Education	funjournal.org	online journal with free access; publishes new methods or tools for neuroscience education at undergrad level; low publishing fees	publishing instrumentation or recording practicals related to neuron electrophysiology; publishing data on effectiveness of activities for improving learning
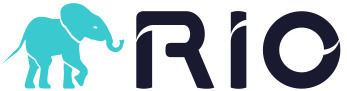	Research Ideas and Outcomes	riojournal.com	open access journal; publishes wide range of products, including research proposals, PhD projects, software descriptions; different peer review options	publishing student work that might otherwise not ’find a home’; review options could give students feedback on projects ideas, proposals, thesis results

## Practicals and course integration

In fourth semester, students in UNAM’s Biomedical Physics undergraduate degree program take a human physiology course, which is divided into modules: (1) nervous, (2) musculoskeletal, (3) biofluids (4) cardiovascular, (5) respiratory (6) gastrointestinal, and (7) renal systems. At present, this class is only lecture. One of our goals with this project was to design hands-on electrophysiology activities to be integrated throughout the course. We briefly describe some of these practicals, where they fit into the course, and how they reinforce concepts from our plan of study. The format of our written documentation accompanying each practical is modeled after BYB’s experiment manual ^64^, with clear learning objectives for before, during, and after practical completion. All our practicals – finished and under development – are at github.com/emckiernan/electrophys, and select ones at electrophys.wordpress.com. A full list of practicals and links to documents, data, and code is available as extended data via Zenodo doi.org/10.5281/zenodo.4540355 ^65^.

### EMG basics: recording from the body’s lever systems

The first practical is designed to teach students the basics of EMG recording, carried out at the end of the musculoskeletal system module. The background written information reinforces physiology concepts seen in class, as well as the application of basic physics concepts seen in other coursework. It begins with a description of how muscle-bone-joint complexes function as lever systems. Students are encouraged to think back to the three types of classical lever system and find corresponding examples of these in the human body. This involves visualizing biomechanics and how the relative position of bones, joints, muscles, and loads will affect movement. The written documentation goes on to reinforce concepts such as how muscle structure affects tension development, length-tension relationships, and the energy requirements for muscle contraction. We then describe the basics of EMG recording, comparing the advantages and disadvantages of invasive versus surface recording, and the basic bipolar differential recording configuration. Study questions prompt students to think about where they will need to place electrodes to record from different muscles and what potential limitations they might encounter.

Students then move on to the experimental phase of the practical where they carry out their own EMG recordings, in groups of 4-6 students depending on class size. Step-by-step instructions on how to perform the recordings are included in the written documentation. However, these are designed to be informative without being too prescriptive and still allowing for exploratory learning. The only requirement is that students record from at least one muscle from each type of lever system, but we do not tell them which muscles to record from or how they should activate these muscles. Students are encouraged to design their own experiments using everyday items available in the classroom or simple exercise aids, like resistance bands or hand grippers, brought from home. Students have performed EMGs from facial muscles while eating, tricep muscles while doing pushups, bicep muscles while arm wrestling or lifting their backpacks, and forearm muscles while performing martial arts movements. Students are also encouraged to explore different types of contraction, including intermittent versus sustained and increasing versus decreasing force.

We have several other EMG practicals still under development, including experimental ones to measure fatigue in the bicep muscle, dual recordings from antagonistic muscle pairs, simultaneous recording of EMG and force sensor measurements from the forearm, and others. In addition, we are developing EMG data analysis practicals. Students will take the recordings they gathered in the first practical, graph them, and learn about the design and application of band-pass and low-pass filters to process their data. They will learn about different techniques used to smooth data, calculate an envelope, and use thresholds to detect start and stop times of muscle contractions. These practicals will be carried out using Jupyter notebooks running Python, thereby simultaneously strengthening students’ programming skills. All practicals are listed in the extended data ( doi.org/10.5281/zenodo.4540355 ^65^).

### ECG basics: recording heart electrical activity before and after exercise

In module 3 of their human physiology course, students learn about the cardiovascular system and carry out a practical to record their ECG before and after exercise. The background written information begins with a description of how the heart performs external mechanical work. Students are encouraged to visualize the heart as a single-chamber pump with inflow and outflow valves, and examine the pressure-volume relationships similar to the way one would with an internal combustion engine ^66^. Students learn about sequential pressure and volume changes in different chambers of the heart during the cardiac cycle, and how to graph this with a pressure-volume loop. The documentation goes on to describe the electrical activity of specialized populations of cells in the heart, including the ionic basis of APs in these cells. Discussing cardiac muscle activity also encourages students to think back to module 2 of the human physiology course when we discussed contraction mechanisms in this muscle type. Finally, we describe the basics of ECG recording, including how the summation of individual potentials leads to the extracellularly recorded events, different recording configurations, and the importance of electrode placement.

Students then move on to the experimental phase, working in groups of 4–6. Volunteers from each group record their ECGs, while other students help with organizing and exporting the data. Students first record their baseline ECG under resting conditions for at least 1–2 minutes. Then, they disconnect the recording device while leaving the electrodes in place and perform light to moderate exercise for at least 5 minutes. After this, students reconnect the device and record their ECG again for at least 1–2 minutes. Students can choose the type of physical activity they perform. For example, students have done push-ups or burpees, ran laps around the building, or gone up and down stairs outside the classroom. We encourage students to compare how different levels of activity change the ECG signal, and how the signal varies across subjects (e.g., athletes versus non-athletes).

We are developing additional experimental practicals designed to explore the relationship between heart and respiratory activity, using simultaneous ECG and spirometry, for example. We are also working on ECG data analysis practicals. Students will take the recordings they gathered during the first ECG practical, graph them, and learn how to detect the peaks of the QRS complex to calculate heart rate and quantify how it changes after different levels of exercise. They will also examine techniques for detecting the P and T waves, and calculating intervals important in clinical evaluations. A full list of these practicals is available in the extended data ( doi.org/10.5281/zenodo.4540355 ^65^).

### Recording accessory muscles during normal and forced respiration

In module 5 of their human physiology course, students learn about the respiratory system, an important part of which is understanding the mechanics of breathing. How do respiratory muscles expand or contract the thoracic cavity and change pressure gradients? How does the participation of different muscles change when respiration is normal versus forced? And to relate back to the musculoskeletal module, how is respiratory muscle contraction related to electrical activity?

In this practical, students record EMGs from the rectus abdominis. This muscle is known as an accessory respiratory muscle because it is not activated during normal exhalation, but is activated during forced exhalation when additional effort is needed to reduce the volume of the thoracic cavity beyond that accomplished by simple elastic recoil ^67^. While recording rectus abdominis EMG, students simultaneously use a spirometer (Vernier) to measure the volume of air moved in and out of the lungs. Students are instructed to perform a sequence of normal breaths interspersed with maximal forced inhalations and exhalations.

The dual recordings allow students to see firsthand that, during normal respiration and forced inhalation, little to no electrical activity is recorded on the EMG because the rectus abdominis is not contracting. However, during forced exhalation, the EMG shows an increase in both the amplitude and frequency of the signal with increased effort and increased volume exhaled. The written materials for the practical are designed to reinforce several physical concepts applied to the study of respiration, including: (1) pressure-volume relationships and Boyle’s Law as applied to the lungs; (2) importance of pressure gradients and Ohm’s Law as applied to airflow; (3) Poiseuille’s Law as applied to measuring airflow through a spirometer, and (4) biomechanics of active lung expansion versus passive elastic recoil. This practical also gives students the opportunity to integrate knowledge from two modules to understand how the musculoskeletal and respiratory systems work together. We are working on developing more practicals that combine electrophysiological recordings with other physiological measurements (e.g., from force, displacement, or gas sensors) to provide similar integrative learning experiences.

## Discussion

### Incorporating electrophysiology into undergraduate education

Less than a decade ago, providing hands-on electrophysiology learning experiences for undergraduates, especially large classes, was not feasible. However, over the last few years, technological advancements have opened up new possibilities for educators. With the introduction of the BYB Neuron SpikerBox in 2011, an easy-to-use, low-cost bioamplifier brought neurophysiology into the classroom ^37^. Since then, the single-channel SpikerBox, and the later two-channel version, have been used to design practicals for undergraduates to record from cricket sensory organs ^68^, grasshopper neurons responding to visual stimuli ^69^, and to study AP conduction velocity in earthworms ^70^. Surveys from these studies indicate that students not only enjoy these hands-on activites, but that they also improve learning outcomes, increasing test scores by as much as 25% on average ^70^. The SpikerBox has even been used as part of a larger program to provide undergraduates the opportunity to teach neuroscience to highschool students ^71^.

In recent years, BYB has released more complex devices for recording ECG, EMG, and single-channel EEG, which have also been used in undergraduate class settings to improve learning. For example, Catena and Carbonneau (2018) describe using the BYB Muscle SpikerBox Pro to record dual-channel EMG as part of an undergraduate biomechanics course ^72^. Their survey results show that students reported “better motivation" and higher “personal responsibility for learning". Test scores for students who had these hands-on learning experiences were also 7% higher compared to students who did not ^72^. Similarly, Judge and colleagues (2020) used BYB equipment to develop ECG and EMG exercises for community college anatomy and physiology courses ^73^. Students who carried out these exercises showed “significant learning gains" ^73^.

Other groups have also developed and shared plans for low-cost electrophysiological recording devices, and in the process created instrumentation exercises for undergraduates. Matsuzaka and colleagues (2012) describe the development of a low-cost (only $85 USD per unit) amplifier for recording EEGs, EMGs, and other electrophysiological signals with students ^74^. Importantly, the authors mention that potential problems of reproducibility and quality control when building these devices “could be resolved if the optimized circuit layout is freely available” (pg. A124). Crisp and colleagues (2016) provide step-by-step instructions for students to build a simple EMG device using a breadboard amplifier, with few components and an assembly time of just 30 minutes ^75^. Wyttenbach and colleagues (2018) review these and other devices as part of a larger discussion on “reducing the cost of electrophysiology in the teaching laboratory” ^76^.

It is not just low cost that is important, but moreso the open approaches taken that have increased the impact of many projects like the ones described above. Sharing hardware schematics and building instructions, openly licensing and publicly documenting code, and growing online support communities – characteristics of projects like Arduino, BYB, and Raspberry Pi – have allowed classrooms and laboratories in limited-resource countries to build capacity ^77^.

### Connections between open approaches

One of the most interesting aspects of this project for us has been working at the intersection of open education and open research, and experiencing firsthand how these approaches can build on one another. Open access, open data, open education, and open source have historically different developments, and are often treated as separate areas of advocacy. However, all these areas share common goals: (1) increased access to information, whether in the form of a textbook, an article, a data set, or code; (2) increased participation, whether in education, research, citizen science, or software development; and (3) better outcomes, whether that means better learning outcomes, more reproducible research, or improved software. How can these open approaches learn from each other and work together to further these goals? In particular, in a limited-resource environment, we wondered whether open educational approaches could also help us build research capacity, and whether open research approaches could also help us create OERs. In our opinion, the answer to both of these questions is a resounding ‘yes’.

The clearest example of this for us was in observing the connections between open education and open source. When we thought about code as not just for research but also an educational resource, it changed how we thought about sharing this product. Previously, we might have simply shared our code as a raw Python file in a GitHub repository, and included some in-line comments and a README file as documentation. However, when we envisioned students or educators reusing our code to learn, we realized it needed more in-depth explanations and exercises. Using Jupyter notebooks, we built up tutorials or lesson plans surrounding the code and transformed these into OERs ^52– 54^. Importantly, after completing these resources we not only had quality OERs to use for our classes, but we also had a bank of well-documented analysis tools to use for future research projects. Organizing and documenting our code in this way may help us with lab group onboarding, as any incoming students can go through the tutorials and quickly get up to speed on our data analysis techniques. Furthermore, in the process of elaborating didactic explanations of our analysis, sometimes we realized ways in which our code could be improved. Therefore, the interaction went both ways: building educational capacity through open practices led to building research capacity and vice versa.

**Figure 2.**
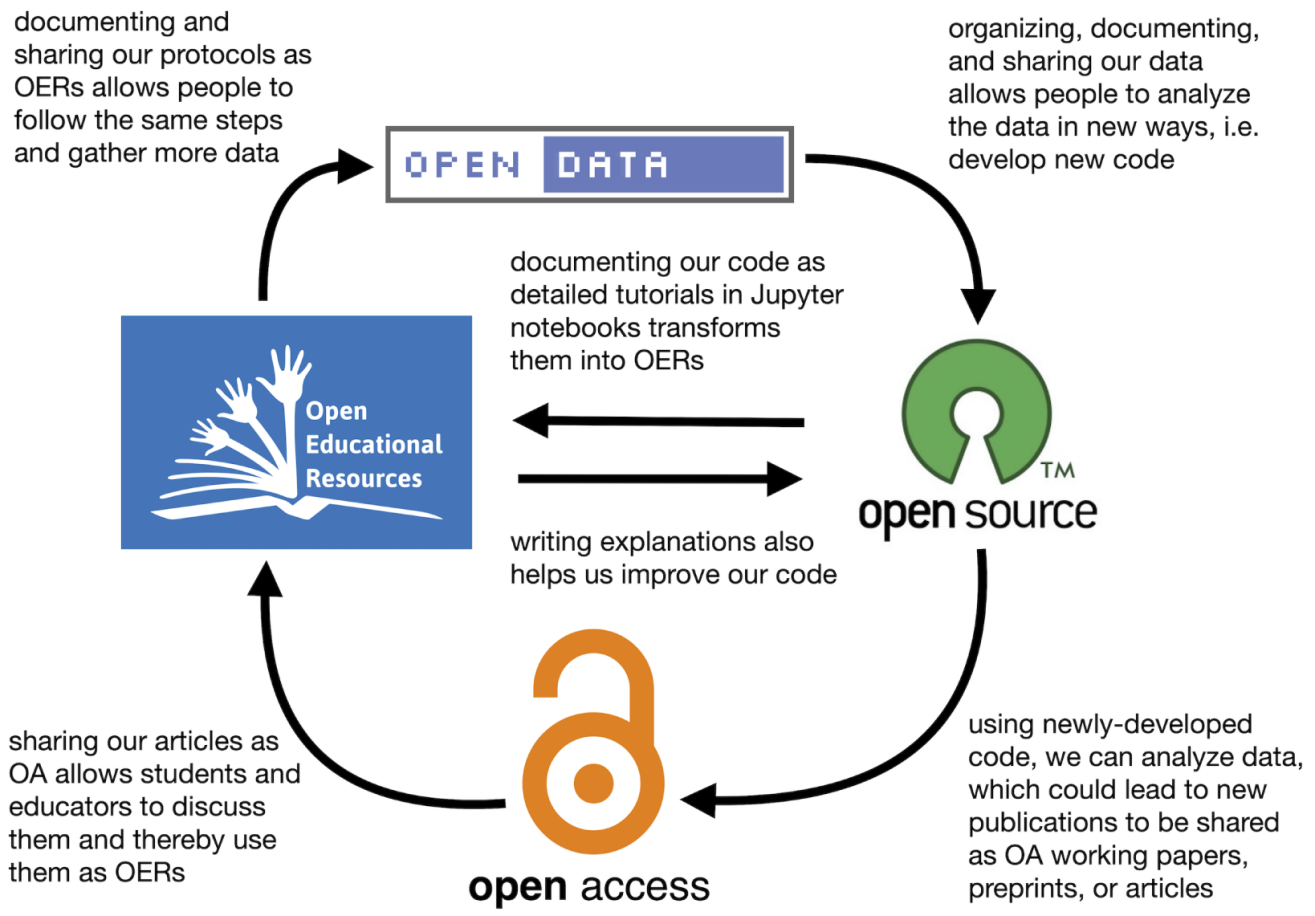
Concept map for how various open approaches connected for us in this project.

We have visually mapped out some of the potential connections between different open approaches ( Figure 2). While this is not an exhaustive map – other open approaches could be included and other connections explored – it is a representation of how these connected for us in this project.

Importantly, one open approach did not automatically lead into the next; there were transformations of the materials and certain conditions that needed to be met at each stage to maintain the flow between each. For example, simply sharing our data would not necessarily allow others to develop new analysis code ( Figure 2, upper right arrow). For this to occur, the data need to be well organized, labelled, and documented, with meta-data included. Admittedly, we are still struggling with the best ways to do this to optimize reuse of our data, and believe this is one area where researchers would benefit from more training. Similarly, shared code does not necessarily become an OER ( Figure 2, central left-pointing arrow). This requires that the code be well explained, often with a surrounding lesson plan and exercises. Open licensing at each stage was also key, since locking down content at any point would stop the flow. However, licensing is different for code, data, and documents. To select licenses for each product, we used resources like the Creative Commons License Chooser ( creativecommons.org/choose) and GitHub’s Choose an Open Source License tool ( choosealicense.com). 

We would also like to encourage researchers to expand their ideas of what they consider an OER. When working with students, there is no clear line where education ends and research begins. The research we do with students, especially with undergraduates, is not necessarily to discover new things but rather to teach students *how* to do research. It is more about the process than the end result, and as such, everything we create during that process – protocols, code, data, notebooks – can potentially be transformed into an OER to train others. We also believe that in thinking of research as a teaching-learning process, with all the documentation and explanations that entails, we may in turn enhance the research itself, improving experimental design and reproducibility.

### Libraries leading in open practice and funding open projects

We are not the first to think about the potential connections or intersections between different open approaches. For years, libraries have been at the forefront of conceptualizing, creating, and managing all kinds of open content ^78– 80^, and thinking about how open practices might connect. The following are all projects led by librarians and information specialists, and/or based in libraries. In 2015, Atenas and Havemann published a book ^81^ arguing that “while Open Data is not always OER, it certainly becomes OER when used within pedagogical contexts” (pg. 22), and presented five case studies where open data were used to teach students programming skills, data literacy, and even promote civic engagement. Elder ^82, 83^ and Walz ^84^ have looked at the differences between open access and open education, but in the process also found areas where these overlap and where they can learn from one another. For the last few years, Virginia Tech libraries has been hosting a series called “Connecting the Opens”, where they invite experts to discuss possible connections between open practices (recordings found at VTechWorks vtechworks.lib.vt.edu). Makerspaces, which often combine aspects of open hardware, open source software, and open education, are increasingly being established and run by libraries ^85– 87^. We hope to see even more of this intersectional work in coming years, and expect that much of it will arise in libraries.

Libraries are also increasingly both leading and funding open scholarship projects, including the development and implementation of OERs. In a 2016 survey of U.S. universities, 64% responded that it was the library who had originated affordable course content (ACC) or OER initiatives at their institutions ^88^. For those with governing bodies overseeing these initiatives, 89% said that libraries were participating members and half said that libraries led the group. Over half of respondents also indicated that funding for ACC/OER initiatives came from library general operating budgets – more than any other institutional or external funding source.

Despite library support for open initiatives, it seems other institutional policies have not necessarily caught up. Walz and colleagues ^88^ write, “survey responses indicate that current university-wide tenure and promotion policies do not explicitly encourage faculty adoption, adaptation, or creation of ACC/OER” (pg. 5). We believe it was important for the success of this project, as well as our own professional development, that our department recognizes and values participation in PAPIME projects in annual performance, promotion, and tenure reviews, and gives us space on evaluation forms to report on non-traditional digital products, including OERs. We encourage institutions to rethink and reform their evaluation policies to incentivize open scholarship, including OER development and adoption, and to seek guidance from libraries on how best to do this. 

## Conclusions

We succeeded in developing a suite of electrophysiology practicals to give biomedical physics undergraduates hands-on training in this important area of study. Our materials are shared as open educational resources to increase their reach and impact. Importantly, open approaches, such as open data, open hardware, and open source software, were crucial to our project’s success, allowing us to maximize limited resources and build both educational and research capacity. We believe open scholarship has a key role to play in the future of undergraduate education, and hope the examples and strategies we have shared here will benefit other educators working to improve learning experiences in the biomedical sciences and beyond.

## Data availability

### Underlying data

Source repository: GitHub. Electrophysiology practicals for undergraduate students. https://github.com/emckiernan/electrophys. 

Archived at time of publication -

Zenodo: electrophys v1.0.1 http://doi.org/10.5281/zenodo.4554420 ^49^

Our GitHub repository, archived via Zenodo, contains all the lesson plans for our electrophysiology practicals, raw electrophysiology data, and data analysis code.

License depends on resource type. Practicals (documents) are shared under the Creative Commons Attribution 4.0 International (CC BY 4.0) license; code under the MIT License; and data (recordings) under the Creative Commons CC0 1.0 Universal license. For more information, see our license file. 

### Extended data

Zenodo. Electrophysiology practicals for undergraduates: links to code, data, and lesson plans (Version v1.0). http://doi.org/10.5281/zenodo.4540355 ^65^

This extended data includes a pdf document with a full list of our practicals (developed and under development) with links to the resources.

Data are available under the terms of the Creative Commons Attribution 4.0 International license (CC-BY 4.0).
