# Supplementary material for: Building capacity through open approaches: Lessons from developing undergraduate electrophysiology practicals
Source: F1000Res. 2021 Mar 8;10:187. [Version 1] doi: 10.12688/f1000research.51049.1 (PMC8600483; doi:10.12688/f1000research.51049.1)
Supplement: Peer review history

# Peer review: Reviewer response for version 1

**DOI:** 10.5256/f1000research.54156.r97262

**Published:** 2021-11-17

**Authors:** Robert Wyttenbach

**Affiliations:** 1 Emory University, Atlanta, Georgia, USA

The authors make a persuasive argument for including electrophysiology (specifically EMG, EEG, and ECG) in a biomedical curriculum. The medical (rather than research) focus and cost justify omitting intracellular recording and concentrating on human subjects.

 The history of their project, while specific to their institution, may be helpful to others in similar positions. However, the core of this report is undoubtedly the open-source equipment, software, and lab exercises. Most of these are linked directly in the text.

 In other educational contexts, invertebrate preparations would be suitable; a few are mentioned in passing. Most of the equipment and software reported here could be used or adapted for such preparations.

Is the work clearly and accurately presented and does it cite the current literature?

Yes

If applicable, is the statistical analysis and its interpretation appropriate?

Not applicable

Are all the source data underlying the results available to ensure full reproducibility?

No source data required

Is the study design appropriate and is the work technically sound?

Yes

Are the conclusions drawn adequately supported by the results?

Yes

Are sufficient details of methods and analysis provided to allow replication by others?

Yes

Reviewer Expertise:

Invertebrate neurophysiology; lab exercise development; software for teaching

I confirm that I have read this submission and believe that I have an appropriate level of expertise to confirm that it is of an acceptable scientific standard.

# Peer review: Reviewer response for version 1

**DOI:** 10.5256/f1000research.54156.r81109

**Published:** 2021-03-25

**Authors:** Javiera Atenas

**Affiliations:** 1 Centre for Excellence in Learning and Teaching, University of Suffolk, Ipswich, UK

The paper presents a solid piece of evidence in Open Educational Practices. It is very well written and it is pedagogically sound. 

 The arguments are well presented, the literature review is clear and informative, as well as adequate, the method is well presented and described, it presents an innovative approach for teaching and learning, thus this paper can have an impact at informing curriculum design, as it provides clear and practical guidelines for the adoption of open data and open educational resources.

Is the work clearly and accurately presented and does it cite the current literature?

Yes

If applicable, is the statistical analysis and its interpretation appropriate?

Not applicable

Are all the source data underlying the results available to ensure full reproducibility?

No source data required

Is the study design appropriate and is the work technically sound?

Yes

Are the conclusions drawn adequately supported by the results?

Yes

Are sufficient details of methods and analysis provided to allow replication by others?

Yes

Reviewer Expertise:

Open Education; Open Data; Curriculum Design

I confirm that I have read this submission and believe that I have an appropriate level of expertise to confirm that it is of an acceptable scientific standard.
